# Silencing of immunoglobulin superfamily containing leucine-rich repeat inhibits gastric cancer cell growth and metastasis by regulating epithelial–mesenchymal transition

**DOI:** 10.1080/21655979.2022.2079303

**Published:** 2022-06-02

**Authors:** Aitao Sun, JinBo Li, Weijing Kong, Xiaodong Jiang

**Affiliations:** a Department of Gastroenterology, Yantaishan Hospital, Yantai, Shandong, P.R. China; b Department of General Surgery, Gaotang County People’s Hospital, Liaocheng, Shandong, P.R. China; c Department of Cardiology, Qingdao Eighth People’s Hospital, Qingdao, Shandong, P.R. China; d Department of Gastrointestinal Surgery, Laizhou People’s Hospital, Yantai, Shandong, P.R. China

**Keywords:** ISLR, gastric cancer, epithelial–mesenchymal transition, invasion

## Abstract

This study aims to investigate the immunoglobulin superfamily containing leucine-rich repeat (ISLR) expression in gastric cancer (GC) and ISLR’s underlying mechanisms regulation of GC progression. Through The Cancer Genome Atlas (TCGA) cohort datasets, we analyzed the ISLR expression in GC tumor tissues and normal tissues. ISLR expression in GC tissues and cells was determined using quantitative real-time polymerase chain reaction. Cell viability, proliferation, migration, and invasion assays were performed in GC cells transfected with sh-ISLR, ISLR plasmids, or controls. TCGA results showed that ISLR expression was higher in GC tumor tissues compared to normal tissues, and its expression levels were related to lymph node metastasis, tumor size, and clinical stage. ISLR was highly expressed in tumor cells. ISLR knockdown suppressed cell viability, proliferation, migration, and invasion in HGC-27 cells, whereas ISLR overexpression led to opposite effects in AGS cells. Gene Set Enrichment Analysis showed that ISLR could activate the epithelial–mesenchymal transition (EMT) signaling pathway. Silencing of ISLR suppressed EMT in HGC-27 cells and overexpression of ISLR promoted EMT in AGS cells. ISLR was overexpressed in both GC cell lines and tumor tissues, and our study first showed that silencing of ISLR inhibited GC cell growth and metastasis by reversing EMT.

## GRAPHICAL ABSTRACT

**Figure:**
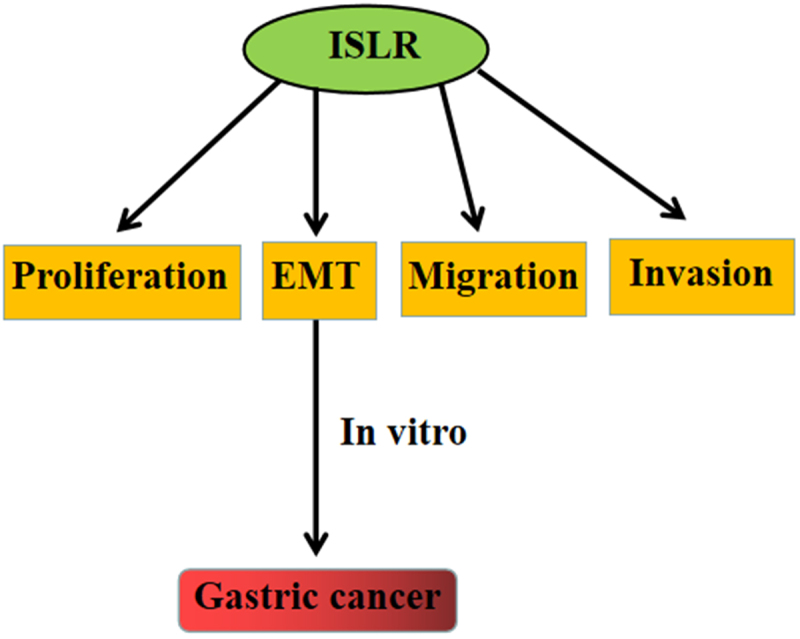

## Highlights

- ISLR expression is up-regulated in gastric cancer tissues and cells.
- ISLR promotes gastric cancer cell proliferation, migration, and invasion.
- ISLR promotes the activation of epithelial–mesenchymal transition.

## Introduction

Gastric cancer (GC) is one of the most common primary malignant tumors, and it is the third principal cause of cancer death, accounting for 9% of fatality in world-wide [1,2]. The incidence and prevalence rates of GC vary by geographical location and are highest in Central and East Asia, Latin America, and Eastern Europe, where 87% of new cases occur, and are much lower in Africa and North America [3,4]. GC is a highly heterogeneous disease with molecular and phenotypic characteristics, and endoscopic resection is the most effective treatment for GC [5,6]. In the last 10 years, the incidence of GC has remarkably decreased, but the incidence rates are still high in many developing countries [7]. Although great progress has been made in the early detection, diagnosis, and treatment of GC, the overall prognosis of GC patients remains poor, and the 5-year survival rate is still about 20.25%, which is mainly because of postoperative recurrence and metastasis of tumor [8,9]. Tumor metastasis and invasiveness are complex and multifactorial process. Therefore, a comprehensive study of the molecular mechanisms that facilitate the evolvement of GC may contribute to improve prevention, diagnosis, and therapy of GC.

The human immunoglobulin superfamily containing leucine-rich repeat (ISLR), a glycosylphosphatidylinositol (GPI)-anchored membrane protein, is a newly discovered family of proteins that covers a leucine-rich repeat (LRR) sequence and immunoglobulin-like domain [10,11]. The ISLR gene with 2.4-kb transcript situates to human chromosome 15q23-q24, and multiple genetic disorders involving this gene have been identified through link analysis [12]. Genome sequencing showed that there were two transcripts of ISLR gene, namely ISLR-1 and ISLR-2, which were stemmed from different first exons 1b or 1a, the expression of ISLR-1 in spinal cord was higher than that of ISLR-2 [10]. Some studies have reported ISLR protein participates in many kinds of biological processes [13–15]. Recently, Kobayashi et al. concluded that ISLR expression was increased in the stroma of patients with colorectal cancer involving colorectal carcinogenesis [13]. In addition, a recent study has reported that ISLR gene takes part in GC occurrence, in which latent molecular mechanism may induce epithelial–mesenchymal transition (EMT) [16]. EMT is a good embryological process and is considered to play a key role in tumor development, including metastasis and invasiveness; thus, cancer cells can obtain more aggressive characteristics [17–19]. However, no study has explored the potential and detailed functional relevance of ISLR and EMT in GC.

In our study, we hypothesized that ISLR may be an important player in GC. Therefore, this work aimed to investigate the biological impact of ISLR on GC cells and its potential mechanisms.

## Materials and methods

### Clinical samples

Tissue samples were collected from 27 GC patients (12 cases stage I/II, 15 cases stage III/IV) from our hospital between May 2017 and December 2019. None of the patients underwent preoperative radiation or chemotherapy. Tissue samples were directly frozen and preserved in liquid nitrogen (−80°C). The study was approved by the Ethics and Research Committees of Yantaishan Hospital (approval number: 201705011), and each patient completed the written informed consent.

### Cell culture

Human GC cell lines (HGC-27, MKN-45, MGC-803, and AGS), normal gastric mucosa epithelial cells (GES-1), and embryonic renal epithelial cell line (293 T) were offered by China Infrastructure of Cell Line Resources (Beijing, China), and were hatched using RPMI‑1640 medium (Thermo Fisher Scientific, Waltham, USA) with 10% fetal bovine serum (FBS) in a 37°C 5% CO_2_ of incubator (Thermo Fisher Scientific). The medium was renewed every 2–3 days [20].

### Cell transfection

Short hairpin RNA against ISLR (sh-ISLR), negative control shRNA (shRNA-NC), and ISLR plasmids were designed and packaged into lentiviral vector by GenePharma (Shanghai, China). According to the manufacturers’ instructions of Lipofectamine 2000 (Invitrogen, Thermo, USA), the 293 T cells were transfected with lentiviral vector and packaging plasmids. After transfection for 48 h, HGC-27 and AGS cells were inoculated and cultivated in 24-well plates for 24 h. Once to 70–80% confluency, 1 × 10^5^ HGC-27 cells were transfected with lentivirus that carries shISLR-1, shISLR-2, or shRNA-NC to establish ISLR downregulated cell model; meanwhile, 1 × 10^5^ AGS cells were transfected with lentivirus carrying ISLR plasmids or vector to construct ISLR-upregulated cells model. Cells with >80% transfection efficiency were used for the subsequent experiments [21].

### Quantitative real-time polymerase chain reaction (qRT-PCR)

RNA was extracted by an RNA kit (Takara, Japan), and it reversed into cDNA via a reverse transcription kit (Takara, Japan). Afterward, cDNA was amplified in a reaction mixture (Applied Biosystems, USA) and 10 μL SYBR-Green qPCR Mix. RT-PCR was conducted on Applied Biosystems 7500 Sequence Detection system (ABI, USA) as follows: predenaturation at 96°C for 3 min; denaturation at 96°C for 15 s; and annealing at 58°C for 30 s, 40 cycles. The primer sequences were as follows: ISLR-forward (F), 5’-GAAGAGGGCCTATTTCCCAT-3’, ISLR-reverse (R), 5’-GCCTTCCATCTGTTGCTGCG-3’; glyceraldehyde-3-phosphate dehydrogenase (GAPDH)-F, 5’-TCCTCTGACTTCAACAGCGACAC-3’; GAPDH-R, 5’-CACCCTGTTGCTGTAGCCAAATTC-3’ [22].

### Cell viability

The cell viability was tested by Cell Counting kit-8 (CCK-8) assay (Beyotime Biotechnology Co., Ltd., Shanghai, China). Approximately 1 × 10^4^ HGC-27 and AGS cells were inoculated and cultured in 96-well plates (100 μL medium/well) at 37°C. Next, the CCK-8 solution (10 μL) was supplemented into per well. The absorbance (450 nm) was detected by Thermomax microplate reader (Thermo Fisher Scientific) at 0, 24, 48, and 72 h to plot the growth curves [23].

### Plate cloning experiment

After 48 h of transfection, according to the concentration of 200 HGC-27 and AGS cells per well, 5 ml cell suspension was inoculated into Petri dishes (diameter 60 mm) and cultured at 37°C and 5% CO_2_ for 2 weeks. The culture medium was replaced with a fresh culture medium every 2–3 days. Once visible colonies appeared in per well, cells were carefully washed twice with PBS, fixed with 4% formaldehyde for 15 min, and stained with 0.05% crystal violet (Beyotime Biotechnology Co., Ltd) for 15 min. After the plates were flushed 3 times with PBS, the colonies with ≥50 cells were directly photographed by cameras and counted by Image J software (Bethesda, MD, USA) [24].

### Wound-healing assay

Firstly, we digested the transfected HGC-27 and AGS cells by trypsin, then 50 μL of cell suspension (5 × 10^5^ cells/ml) was added into per well of a 6-well plate. Then, once the fusion degree of cells achieved 80%, a 200 µL pipette tip was used to draw a horizontal line in per well. Finally, the cell movement was observed and photographed by a microscope (Olympus Corp.) at 48 h after scratching to evaluate healing rate [25].

### Transwell assay

The cell invasiveness and migration capabilities were assessed by 8 μm porous membranes of transwell chambers coated with or without Matrigel (BD Biosciences, NJ, USA). 5 × 10^5^ cells/mL of HGC-27 or AGS cells cultured in RPMI-1640 medium was seeded onto the upper chamber of the Transwell insert. In the lower chamber, RPMI-1640 medium containing 10% FBS was supplemented. 48 h later, cells were flushed with PBS for 3 times, fixed with 4% paraformaldehyde, and stained with 0.2% crystal violet (Beyotime Biotechnology Co., Ltd). Finally, the images were captured by an inverted microscope (Nikon, Tokyo, Japan) [26].

### Western blotting

Cell and tissue samples were lysed using RIPA lysis buffer (Beyotime Biotechnology Co., Ltd) for 30 min. The proteins were separated by 10% sodium dodecyl sulfate-polyacrylamide gel electrophoresis. The specific protein was transferred to a polyvinylidene fluoride membrane. After blocking with 5% nonfat dry milk for 30 min, specific proteins were incubated with primary antibodies [ISLR, GAPDH, Snail, Twist, E-cadherin, Vimentin, N-cadherin, biglycan (BGN), matrix metalloproteinase (MMP)-2, and secreted frizzled related protein 4 (SFRP4); 1:1000, Sigma, USA] overnight at 4°C. After three washes with Tris-buffered saline with 0.1% Tween 20 detergent, the membranes were incubated with horseradish peroxidase conjugated secondary antibodies for 1 h. Finally, enhanced chemiluminescence reagents (GE Healthcare, WI, USA) were used to visualize the protein signals [27].

### The Cancer Genome Atlas (TCGA) data for human GC

The relation between ISLR expression and cancer stages was retrieved from UALCAN (http://ualcan.path.uab.edu/) by analyzing the clinicopathological data available from the TCGA project for human GC. The Kaplan–Meier method was performed to analyze the underlying function of ISLR on overall survival (OS) rates of GC patients [28].

*Gene set enrichment analysis* (GSEA)

GSEA was performed to analyze the gene set associated with ISLR in GC. According to the median expression value, the expression of ISLR was set to high and low. The related genes and pathways of ISLR were researched in c2.cp.kegg.v7.0.symbols.gmt dataset through GSEA v3 software. Falseovery rate less than 0.25 was used to identify significantly enriched genes [29].

### Statistical analysis

All data were analyzed using GraphPad Prism 7 (USA). All tests were made in triplicate. Data are presented as the mean ± standard deviation. Differences between the two groups and among multiple groups were assessed using Student’s t-test and analysis of variance, respectively. The relationship between the ISLR expression and the clinicopathological characteristics of GC patients was estimated by chi-square test. Statistical significance was set at P < 0.05.

## Results

In this study, we investigated the influence of ISLR on the biological behavior of GC cells and its potential mechanisms. Our findings showed that ISLR promoted GC cell proliferation, migration, and invasion by regulating the EMT pathway.

### ISLR is upregulated in GC patients

**Figure 1.**
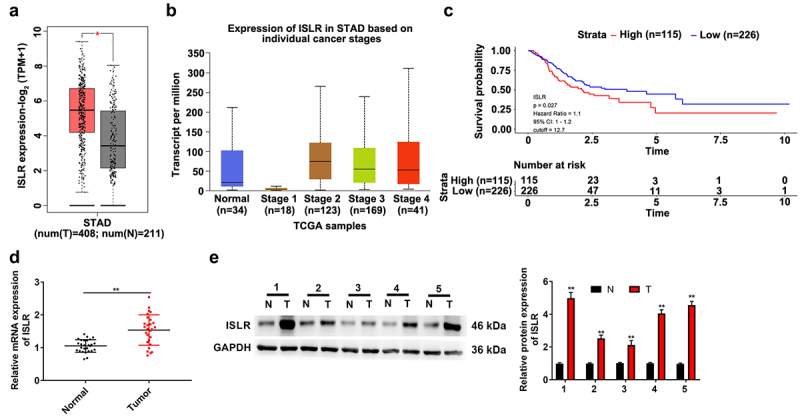
ISLR was up-regulated expression in gastric cancer. (a) The TCGA-STAD datasets indicated ISLR expression in GC tumor tissues was higher compared with that in normal tissues. (b) The ISLR expression in normal tissues and stage 1/2/3/4 of STAD. (c) As Kaplan–Meier survival plots shown, the higher ISLR abundance had relation to a poorer overall survival. (d) The mRNA expression of ISLR in tumor tissues and adjacent normal tissues of 27 patients with gastric cancer was detected using qRT-PCR. (e) Western blotting was used to detect the protein expression of ISLR in tumor tissues and adjacent normal tissues from 5 patients with gastric cancer. **P < 0.01, compared with normal tissues. T, gastric cancer tissue; N, adjacent normal tissues.

To evaluate the ISLR expression in GC, we analyzed the TCGA stomach adenocarcinoma (STAD) cohort datasets. Our results indicated that the ISLR expression in GC tumor tissues was higher compared with that in normal tissues (Figure 1a). In addition, ISLR expression in patients with stage 1 was decreased when confronted with that in normal tissues, while ISLR expression in patients with stage 2/3/4 significantly increased (Figure 1b). Additionally, according to the expression level of the ISLR in GC cases in the TCGA-STAD cohort, OS analysis was performed using the Kaplan–Meier method. A group cutoff of ‘quartile’ was used to divide samples as high- or low-expression group. Low-expression group includes 39 stage I patients, 64 stage II patients, 92 stage III patients, 25 stage IV patients, and 6 unknown stage. High-expression group includes 8 stage I patients, 42 stage II patients, 46 stage III patients, 10 stage IV patients, and 9 unknown stage. As shown in Figure 1c, GC patients with high ISLR expression had poorer OS than that of GC patients with low ISLR expression (hazard ratio = 1.1, 95% CI: 1–1.2, p = 0.027). Then, we detected ISLR expression in 27 GC patients through qRT-PCR and found that GC tissues showed significantly higher ISLR expression than that in normal tissues (Figure 1d). The protein level of ISLR was higher in tumor tissues than in normal tissues of five GC patients, as detected by western blotting (Figure 1e). To explore the clinical value of ISLR expression in GC, we analyzed the correlations between the mRNA expression of ISLR and the clinicopathological characteristics of GC patients. Based on the median value of ISLR expression, we divided GC patients into high ISLR expression group (n = 13) and low ISLR expression group (n = 14). As shown in Table 1, ISLR expression was correlated with tumor size, lymph node metastasis, and clinical stage, while ISLR expression had no relation to age or gender.

**Table 1.** Correlations between ISLR expression and clinicopathological characteristics of gastric cancer

Characteristics	Number	ISLR expression		P value
		low	high	
Age (years)				
<60	10	4	6	0.516
≥60	17	9	8	
Gender				
Male	19	9	10	0.901
Female	8	4	4	
Tumor size (cm)				
<3	7	6	1	0.021*
≥3	20	7	13	
Clinical stage				
I/II	12	9	3	0.013*
III/IV	15	4	11	
Lymph node metastasis				
Absent	9	7	2	0.029*
Present	18	6	12

### ISLR silencing suppresses the proliferation of GC cells

**Figure 2.**
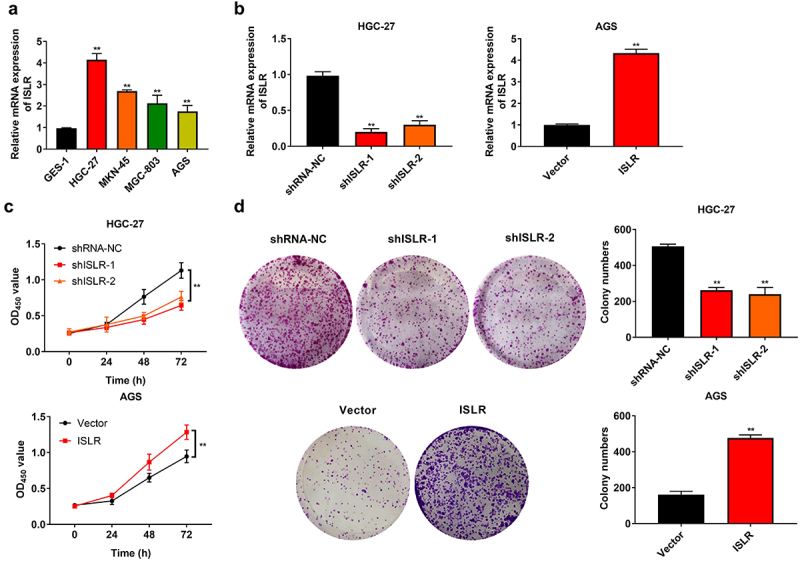
ISLR silencing suppressed gastric cancer cell proliferation. (a) The mRNA expression of ISLR in human gastric cancer cell lines (HGC-27, MKN-45, MGC-803, and AGS) and normal human gastric mucosa epithelial cells (GES-1) was tested using qRT-PCR. (b) HGC-27 cells were transfected with ShISLR-1, ShISLR-2, or ShRNA-NC and AGS cells were transfected with ISL*R plasmid*s or vector, then the mRNA expression of ISLR was examined in HGC-27 and AGS cells using qRT-PCR. (c) Cell viability of HGC-27 and AGS cells was evaluated using CKK8 assay. (d) Cell proliferation of HGC-27 and AGS cells was evaluated using colony formation assay. **P < 0.01, compared with GES-1, shRNA-NC, or Vector group.

The mRNA expression of ISLR in different GC cell lines was determined by qRT-PCR. The results suggested that ISLR was highly expressed in tumor cell lines (HGC-27, MKN-45, MGC-803, and AGC cells), with the expression in HGC-27 cells being the most pronounced and that in AGS cells being the lowest (Figure 2a). Afterward, ISLR was silenced in HGC-27 cells and overexpressed in AGS cells, which was certified by qRT-PCR (Figure 2b). The CKK-8 and colony formation assays clearly manifested that ISLR silencing prohibited the viability and proliferation of HGC-27 cells, nevertheless ISLR overexpression facilitated the viability and proliferation of AGS cells (Figure 2(c-d)).

### ISLR silencing suppresses the migration and invasion of GC cells

**Figure 3.**
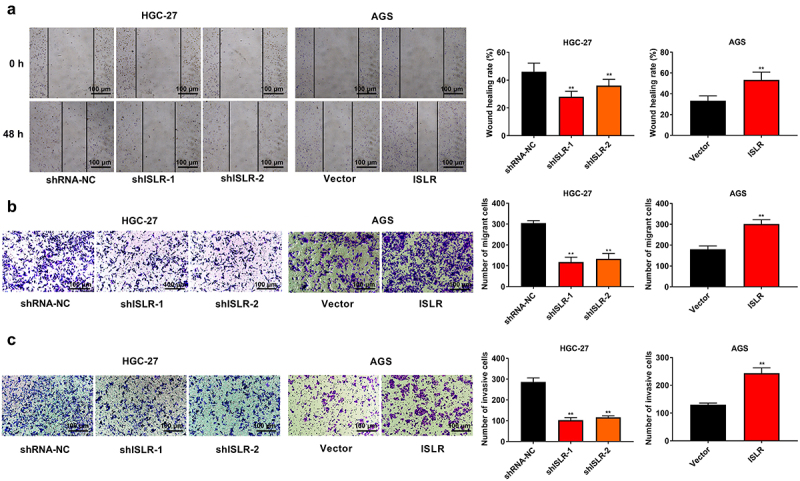
ISLR silencing suppressed migration and invasion in gastric cancer cells. (a) Wound healing was used to detect cell migration in HGC-27 and AGS cells. (b) Transwell assay was used to detect cell migration in HGC-27 and AGS cells. (c) Transwell assay was performed to detect cell invasion in HGC-27 and AGS cells. **P < 0.01, compared with shRNA-NC or Vector group.

Wound healing and transwell assay was carried out to determine the role of ISLR in GC cell metastasis and invasiveness. ISLR silencing evidently weakened the adhesion ability of HGC-27 cells, whereas ISLR overexpression had the opposite effect on AGS cells (Figure 3a). ISLR downregulation obviously restrained cell migration and invasion in HGC-27 cells, while the opposite effect was confirmed in AGS cells with ISLR overexpression (Figure 3(b-c)).

### ISLR promotes EMT activation

**Figure 4.**
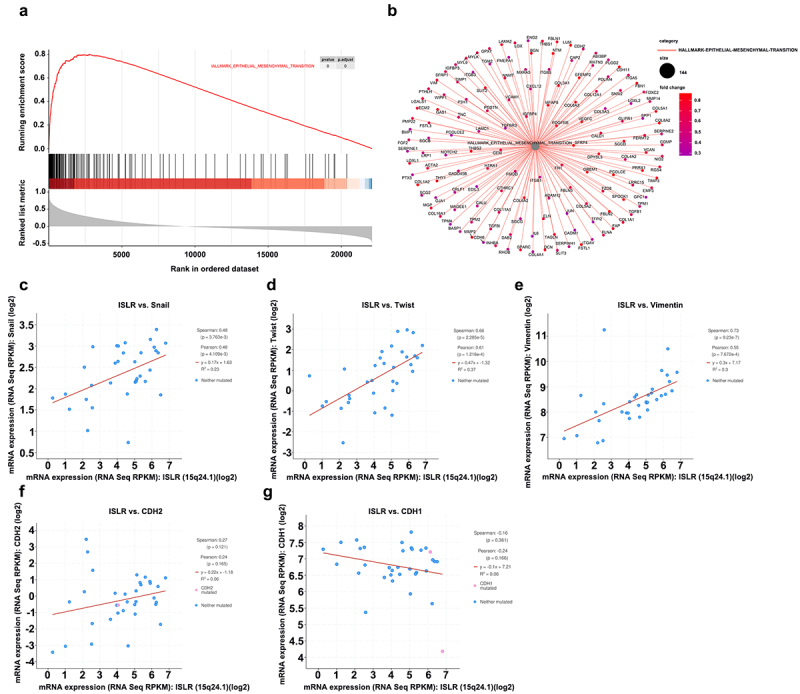
ISLR promotes EMT activation. (a) GESA analysis showed that ISLR regulated HALLMARK_EPITHELIAL_MESENCHYMAL_TRANSITION signaling pathway. (b) EMT downstream enrichment genes. (c-g) According to TCGA database, the expression correlation between ISLR gene and EMT markers [Snail, Twist, Vimentin, N-cadherin (also known as CDH2), and E-cadherin (also known as CDH1)] was analyzed.

To probe the latent mechanisms of ISLR in GC progression, the KEGG downstream pathway of ISLR was evaluated by GSEA. The findings showed that ISLR expression could activate the EMT signaling pathway (Figure 4a). Furthermore, many EMT downstream enrichment genes were obtained (Figure 4b). Based on TCGA database, the expression correlation between ISLR gene and EMT markers was analyzed. We found that the ISLR expression was positively correlated with the expression of Snail, Twist, Vimentin, and N-cadherin (also known as CDH2) (Figure 4(c-f)), while ISLR expression was inversely correlated with E-cadherin (also known as CDH1) expression (Figure 4g).

### ISLR silencing suppresses EMT

**Figure 5.**
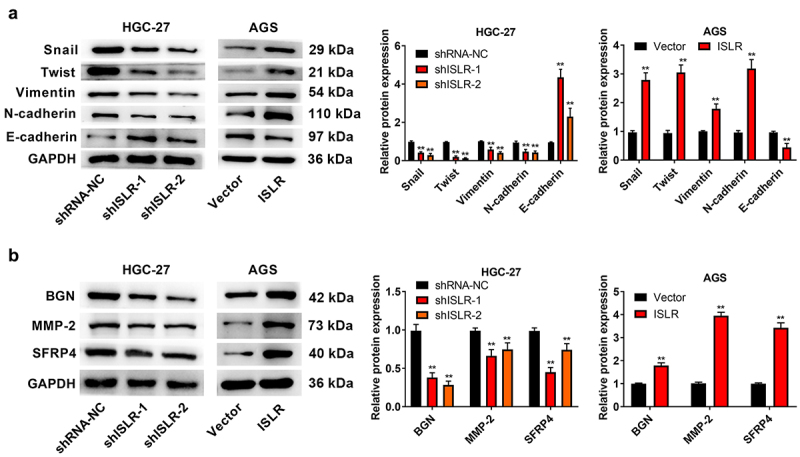
ISLR silencing suppressed EMT signaling pathway. (a) Western blot assay was applied to detect the expression of EMT-related proteins (Snail, Twist, Vimentin, N-cadherin, and E-cadherin) in HGC-27 and AGS cells. (b) Western blot assay was used to detect the protein expression of EMT pathway downstream enrichment genes (BGN, MMP-2, and SFRP4) in HGC-27 cells and AGS cells. **P < 0.01, compared with shRNA-NC or Vector group.

The changes of expression level of proteins related to EMT signaling pathway and some EMT downstream enrichment genes were evaluated by western blotting. Just as expected, ISLR silencing led to a statistically remarkable increase in E-cadherin protein expression and resulted in a dramatic decrease in the expression of Snail, Twist, Vimentin, and N-cadherin, and BGN, MMP-2, and SFRP4 in HGC-27 cells. Rather, the overexpressed ISLR had a counter influence on the expression of those proteins in AGS cells (Figure 5(a-b)). Thus, ISLR silencing suppresses EMT in GC.

## Discussion

GC is usually diagnosed at advanced stage, and recurrence and metastasis are the greatest challenges in the treatment process [30]. Although environmental factors, such as *Helicobacter pylori* infection, smoking, and diet, have been proven to be risk factors, host genetics also play a key role in GC occurrence [31,32]. The multiformity and role of host genetics in the transcriptional active part of the genome and abnormalities in a number of cancers, including GC, have been confirmed in many studies, prompting research to identify molecular candidates for diagnosis and treatment [33]. In this research, we found that ISLR expression in GC tissues was higher than that in normal tissues. In addition, ISLR expression was correlated with tumor size, lymph node metastasis, and clinical stage. Collectively, these findings suggest the potential impact of ISLR on the etiology of GC.

ISLR is detected in muscle tissues and satellite cells, and regulates the muscle regeneration and muscle atrophy [34,35]. Cui et al. have reported that ISLR plays a significant role in the differentiation of myoblasts, alleviating skeletal muscle atrophy and preventing myocyte apoptosis through IGF/PI3K/AKT-FOXO signaling pathway [34]. Xu et al. and Kobayashi et al. have verified that ISLR is crucial for regulating the growth of epithelial cells during intestinal tumourigenesis, particularly in colorectal carcinoma [36,37]. In our study, the results showed that ISLR silencing inhibited cell proliferation, migration, and invasion in HGC-27 cells, whereas ISLR overexpression in AGS cells had the opposite effects.

In recent years, numerous molecular markers, including miRNAs, long noncoding RNAs, and oncogenic ncRNAs, involved in EMT have been shown to manage most cellular processes, leading to the development of many cancers, including GC [3,38–42]. In clinical practice, EMT is associated with a poor prognosis in cancer patients [43]. Invasion and metastasis of tumor cells are the primary causes of death in patients with GC. Accumulating evidences indicates that EMT plays an important role in intercellular adhesion, metastasis, and invasiveness, and is closely related to the adverse clinical outcomes of GC patients [44]. In 2020, Shu et al. reported that ISLR expression was relevant to the activation of the EMT pathway, according to GSCAlite pathway score analysis [16]. In our study, GSEA showed that ISLR could activate the EMT signaling pathway. The expression of collagen, vimentin, fibronectin, and N-cadherin was increased, and expression of E-cadherin was reduced in EMT, leading to cell metastasis and invasiveness [44]. The transcription regulators such as Slug, β-catenin, Snail, and Twist were up-regulated in EMT [45]. MMPs are a class of zinc containing enzymes that can decrease extracellular matrix and promote cell invasion. BGN, as a constituent of ECM, is considered a new mesenchymal marker for the EMT, and is overexpressed in GC tissues and expedited metastasis [46]. SFRP4 can drive GC invasion and is an early predictor of GC recrudescence [47]. Our findings indicated that ISLR silencing led to a remarkable upregulation of E-cadherin and downregulation of Snail, Twist, Vimentin, and N-cadherin, as well as EMT downstream enrichment genes (BGN, MMP-2, and SFRP4). However, the overexpressed ISLR had contrary influence on the expression of those proteins.

## Conclusion

We observed that ISLR expression was increased in both GC cells and tumor tissues, and our study, for the first time, indicated that ISLR silencing inhibited cell growth and metastasis by regulating the EMT pathway. Thus, SLR silencing expression may have important implications in GC therapy. Our results provided evidence that ISLR induces EMT and promotes invasion and metastasis in GC cells.

## Supplementary Material

Supplemental Material

## Data Availability

The datasets used and analyzed during the current study are available from the corresponding author on reasonable request.

## References

[1] Smyth EC, Nilsson M, Grabsch HI, et al. Gastric cancer. Lancet. 2020;396(10251):635–648. 32861308 10.1016/S0140-6736(20)31288-5

[2] Petryszyn P, Chapelle N, Matysiak-Budnik T. Gastric cancer: where are we heading? Dig Dis. 2020;38(4):280–285. 32062657 10.1159/000506509

[3] Bure IV, Nemtsova MV, Zaletaev DV. Roles of E-cadherin and noncoding RNAs in the epithelial-mesenchymal transition and progression in gastric cancer. Int J Mol Sci. 2019;20 (12): 2870. 10.3390/ijms20122870 PMC6627057 31212809

[4] Wang J, Zhang M, Hu X, et al. miRNA-194 predicts favorable prognosis in gastric cancer and inhibits gastric cancer cell growth by targeting CCND1. FEBS Open Bio. 2021;11(7):1814–1826. 10.1002/2211-5463.13125 PMC8255842 33605558

[5] Rawla P, Barsouk A. Epidemiology of gastric cancer: global trends, risk factors and prevention. Prz Gastroenterol. 2019;14(1):26–38. 30944675 10.5114/pg.2018.80001 PMC6444111

[6] Johnston FM, Beckman M. Updates on management of gastric cancer. Curr Oncol Rep. 2019;21(8):67. 31236716 10.1007/s11912-019-0820-4

[7] Camargo MC, Figueiredo C, Machado JC. Review: gastric malignancies: basic aspects. Helicobacter. 2019;24(S1):e12642. 31486241 10.1111/hel.12642

[8] Suzuki H, Oda I, Abe S, et al. High rate of 5-year survival among patients with early gastric cancer undergoing curative endoscopic submucosal dissection. Gastric Cancer. 2016;19(1):198–205. 25616808 10.1007/s10120-015-0469-0

[9] Li D, Cheng P, Wang J, et al. IRF6 is directly regulated by ZEB1 and ELF3, and predicts a favorable prognosis in gastric cancer. Front Oncol. 2019;9:220. 31019894 10.3389/fonc.2019.00220 PMC6458252

[10] Nagasawa A, Kudoh J, Noda S, et al. Human and mouse ISLR (immunoglobulin superfamily containing leucine-rich repeat) genes: genomic structure and tissue expression. Genomics. 1999;61(1):37–43. 10512678 10.1006/geno.1999.5934

[11] Nagasawa A, Kubota R, Imamura Y, et al. Cloning of the cDNA for a new member of the immunoglobulin superfamily (ISLR) containing leucine-rich repeat (LRR). Genomics. 1997;44(3):273–279. 9325048 10.1006/geno.1997.4889

[12] Lind L, Sandstrom H, Wahlin A, et al. Localization of the gene for congenital dyserythropoietic anemia type III, CDAN3, to chromosome 15q21-q25. Hum Mol Genet. 1995;4(1):109–112. 7711721 10.1093/hmg/4.1.109

[13] Homma S, Shimada T, Hikake T, et al. Expression pattern of LRR and Ig domain-containing protein (LRRIG protein) in the early mouse embryo. Gene Expression Patterns GEP. 2009;9(1):1–26. 18848646 10.1016/j.gep.2008.09.004

[14] Yoon IK, Kim HK, Kim YK, et al. Exploration of replicative senescence-associated genes in human dermal fibroblasts by cDNA microarray technology. Exp Gerontol. 2004;39(9):1369–1378. 15489060 10.1016/j.exger.2004.07.002

[15] Lugowska A, Hetmanczyk-Sawicka K, Iwanicka-Nowicka R, et al. Gene expression profile in patients with gaucher disease indicates activation of inflammatory processes. Sci Rep. 2019;9(1):6060. 30988500 10.1038/s41598-019-42584-1 PMC6465595

[16] Li S, Zhao W, Sun M. An analysis regarding the association between the ISLR gene and gastric carcinogenesis. Front Genet. 2020;11:620. 32612640 10.3389/fgene.2020.00620 PMC7308588

[17] Yue B, Song C, Yang L, et al. METTL3-mediated N6-methyladenosine modification is critical for epithelial-mesenchymal transition and metastasis of gastric cancer. Mol Cancer. 2019;18(1):142. 31607270 10.1186/s12943-019-1065-4 PMC6790244

[18] Huang X, Chen C, Xu Y, et al. Infiltrating T-cell abundance combined with EMT-related gene expression as a prognostic factor of colon cancer. Bioengineered. 2021;12(1):2688–2701. 34180352 10.1080/21655979.2021.1939618 PMC8806648

[19] Cui X, Shan T, Qiao L. Collagen type IV alpha 1 (COL4A1) silence hampers the invasion, migration and epithelial–mesenchymal transition (EMT) of gastric cancer cells through blocking Hedgehog signaling pathway. Bioengineered. 2022;13(4):8972–8981. 35297303 10.1080/21655979.2022.2053799 PMC9161915

[20] Wang X, Li Y, Fang Z, et al. Elevated expression of NFE2L3 promotes the development of gastric cancer through epithelial-mesenchymal transformation. Bioengineered. 2021;12(2):12204–12214. 34783304 10.1080/21655979.2021.2005915 PMC8810066

[21] Dalby B, Cates S, Harris A, et al. Advanced transfection with Lipofectamine 2000 reagent: primary neurons, siRNA, and high-throughput applications. Methods. 2004;33(2):95–103. 15121163 10.1016/j.ymeth.2003.11.023

[22] Livak KJ, Schmittgen TD. Analysis of relative gene expression data using real-time quantitative PCR and the 2−ΔΔCT method. Methods. 2001;25(4):402–408. 11846609 10.1006/meth.2001.1262

[23] Yang J, Fan L, Liao X, et al. CRTAC1 (Cartilage acidic protein 1) inhibits cell proliferation, migration, invasion and epithelial-mesenchymal transition (EMT) process in bladder cancer by downregulating Yin Yang 1 (YY1) to inactivate the TGF-β pathway. Bioengineered. 2021;12(2):9377–9389. 34818994 10.1080/21655979.2021.1974645 PMC8809913

[24] Zhao N, Wang C, Guo P, et al. CCDC106 promotes the proliferation and invasion of ovarian cancer cells by suppressing p21 transcription through a p53-independent pathway. Bioengineered. 2022;13(4):10957–10973. 10.1080/21655979.2022.2066759 PMC9208459 35484984

[25] Yu Y, Wang W, Lu W, et al. Inhibin β-A (INHBA) induces epithelial-mesenchymal transition and accelerates the motility of breast cancer cells by activating the TGF-β signaling pathway. Bioengineered. 2021;12(1):4681–4696. 34346300 10.1080/21655979.2021.1957754 PMC8806747

[26] Zhong X, Cai Y. Long non-coding RNA (lncRNA) HOXD-AS2 promotes glioblastoma cell proliferation, migration and invasion by regulating the miR-3681-5p/MALT1 signaling pathway. Bioengineered. 2021;12(2):9113–9127. 34802389 10.1080/21655979.2021.1977104 PMC8810070

[27] Wang J-S, Wang M-J, Lu X, et al. Artesunate inhibits epithelial-mesenchymal transition in non-small-cell lung cancer (NSCLC) cells by down-regulating the expression of BTBD7. Bioengineered. 2020;11(1):1197–1207. 33108235 10.1080/21655979.2020.1834727 PMC8291784

[28] Therneau TM, Lumley T. Package ‘survival’. R Top Doc. 2015;128:28–33.

[29] Aea S. Gene set enrichment analysis: a knowledge-based approach for interpreting genome-wide expression profiles. Proc Natl Acad Sci U S A. 2005;102(43):15545–15550. 16199517 10.1073/pnas.0506580102 PMC1239896

[30] Wu CE, Xue WW, Zhuang YW, et al. A clinical study on the efficacy of Yiqi Huayu Jiedu decoction for reducing the risk of postoperative recurrence and metastasis of gastric cancer: protocol for a multicenter, randomized, double-blind, placebo-controlled trial. Medicine (Baltimore). 2020;99(48):e23417. 33235121 10.1097/MD.0000000000023417 PMC7710168

[31] Sandoval-Borquez A, Saavedra K, Carrasco-Avino G, et al. Noncoding genomics in gastric cancer and the gastric precancerous cascade: pathogenesis and biomarkers. Dis Markers. 2015;2015:503762. 26379360 10.1155/2015/503762 PMC4563069

[32] Jia ZF, Zhang SL, Cao XY, et al. Interaction between *Helicobacter pylori* and host genetic variants in gastric carcinogenesis. Future Oncol. 2016;12(18):2127–2134. 27324311 10.2217/fon-2016-0233

[33] Tanikawa C, Kamatani Y, Toyoshima O, et al. Genome-wide association study identifies gastric cancer susceptibility loci at 12q24.11-12 and 20q11.21. Cancer Sci. 2018;109(12):4015–4024. 30281874 10.1111/cas.13815 PMC6272082

[34] Cui C, Han S, Shen X, et al. ISLR regulates skeletal muscle atrophy via IGF1-PI3K/Akt-Foxo signaling pathway. Cell Tissue Res. 2020;381(3):479–492. 32696215 10.1007/s00441-020-03251-4

[35] Zhang K, Zhang Y, Gu L, et al. Islr regulates canonical Wnt signaling-mediated skeletal muscle regeneration by stabilizing dishevelled-2 and preventing autophagy. Nat Commun. 2018;9(1):5129. 30510196 10.1038/s41467-018-07638-4 PMC6277414

[36] Xu J, Tang Y, Sheng X, et al. Secreted stromal protein ISLR promotes intestinal regeneration by suppressing epithelial Hippo signaling. EMBO J. 2020;39(7):e103255. 32128839 10.15252/embj.2019103255 PMC7110107

[37] Kobayashi H, Gieniec KA, Wright JA, et al. The balance of stromal BMP signaling mediated by GREM1 and ISLR drives colorectal carcinogenesis. Gastroenterology. 2020;160 (4): 1224–1239. 33197448 10.1053/j.gastro.2020.11.011 PMC7617122

[38] Choi RS, Lai WYX, Lee LTC, et al. Current and future molecular diagnostics of gastric cancer. Expert Rev Mol Diagn. 2019;19(10):863–874. 31448971 10.1080/14737159.2019.1660645

[39] Tam C, Wong JH, Tsui SKW, et al. LncRNAs with miRNAs in regulation of gastric, liver, and colorectal cancers: updates in recent years. Appl Microbiol Biotechnol. 2019;103(12):4649–4677. 31062053 10.1007/s00253-019-09837-5

[40] Li D, Wang J, Zhang M, et al. LncRNA MAGI2-AS3, regulated by BRD4, is an independent prognostic marker and promotes gastric cancer progression via maintaining ZEB1 overexpression. Mol Ther Nucleic Acids. 2019;19:109–123. 31837602 10.1016/j.omtn.2019.11.003 PMC6920306

[41] Li D, She J, Hu X, et al. The ELF3-regulated lncRNA UBE2CP3 is over-stabilized by RNA–RNA interactions and drives gastric cancer metastasis via miR-138-5p/ITGA2 axis. Oncogene. 2021;40(35):1–13. 34274947 10.1038/s41388-021-01948-6 PMC8413130

[42] Li D, Xu M, Wang Z, et al. The EMT-induced lncRNA NR2F1-AS1 positively modulates NR2F1 expression and drives gastric cancer via miR-29a-3p/VAMP7 axis. Cell Death Dis. 2022;13(1): 022–04540. 10.1038/s41419-022-04540-2 PMC8791943 35082283

[43] Li G, Su Q, Liu H, et al. Frizzled7 promotes epithelial-to-mesenchymal transition and stemness via activating canonical Wnt/beta-catenin pathway in gastric cancer. Int J Biol Sci. 2018;14(3):280–293. 29559846 10.7150/ijbs.23756 PMC5859474

[44] Xu G, Meng L, Yuan D, et al. MEG3/miR21 axis affects cell mobility by suppressing epithelial mesenchymal transition in gastric cancer. Oncol Rep. 2018;40(1):39–48. 29749532 10.3892/or.2018.6424 PMC6059753

[45] Fioroni I, Dell’Aquila E, Pantano F, et al. Role of c-mesenchymal-epithelial transition pathway in gastric cancer. Expert Opin Pharmacother. 2015;16(8):1195–1207. 25881479 10.1517/14656566.2015.1037739

[46] Hu L, Zang MD, Wang HX, et al. Biglycan stimulates VEGF expression in endothelial cells by activating the TLR signaling pathway. Mol Oncol. 2016;10(9):1473–1484. 27590684 10.1016/j.molonc.2016.08.002 PMC5423211

[47] Busuttil RA, George J, House CM, et al. SFRP4 drives invasion in gastric cancer and is an early predictor of recurrence. Gastric Cancer. 2020;24(3):589–601. 33277667 10.1007/s10120-020-01143-8 PMC8064978

