# Supplementary material for: Silencing of immunoglobulin superfamily containing leucine-rich repeat inhibits gastric cancer cell growth and metastasis by regulating epithelial–mesenchymal transition
Source: Bioengineered. 2022 Jun 2;13(5):13544–54. doi: 10.1080/21655979.2022.2079303 (PMC9276042; doi:10.1080/21655979.2022.2079303)
Supplement: Supplemental Material [file KBIE_A_2079303_SM6012.zip › Ethical approval.docx]

**烟台市烟台山医院伦理委员会快速伦理审查批件**

伦理号（201705011）

| 项目名称 | **Silencing of** **ISLR** **inhibits** **gastric cancer cells growth and metastasis by** **regulating epithelial-mesenchymal transition** | | |
| --- | --- | --- | --- |
| 项目负责人 | 孙爱涛 | 承担科室 | 消化内科 |
| 项目类型 | 研究者发起的研究项目 | | |
| 审查文件 | 1.伦理审查申请表；  2.研究方案；  3.知情同意书；  4.主要研究者简历。 | | |
| 审查时间 | 2017年5月11日 | 审查方式 | 快速审查 |
| 伦理审查意见 | | 同意 | |
| 审批意见  研究者的资格、经验符合试验要求;研究方案符合科学性和伦理原则的要求;获得知情同意的方法适当;受试者可能遭受的风险程度与研究预期的受益相比合适。 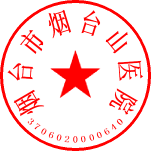 同意开展该项目的研究！  烟台市烟台山医院动物研究伦理委员会  （盖章）  2017年5月11日 | | | |
